# Opioid-free anesthesia—dexmedetomidine as adjuvant in erector spinae plane block: a case series

**DOI:** 10.1186/s13256-021-02868-5

**Published:** 2021-05-29

**Authors:** Antonio Coviello, Danilo Esposito, Roberta Galletta, Alfredo Maresca, Giuseppe Servillo

**Affiliations:** Department of Anesthesiology and Intensive Care Medicine, Policlinico - Federico II University Hospital, 80100 Naples, Italy

**Keywords:** Case report, Dexmedetomidine, Multimodal analgesia, Opioid-free anesthesia, US-ESPB

## Abstract

**Background:**

Laparoscopic pain is related to the stretching of the peritoneum and peritoneal irritation caused by insufflation of the parietal peritoneum with carbon dioxide. In 2017, erector spinae plane block (ESPB) was described for management of postoperative pain following open and laparoscopic abdominal surgery. The use of multimodal anesthesia reduces both intraoperative and postoperative opioid use and improves analgesia. The addition of dexmedetomidine to the anesthetic mixture significantly prolongs analgesia, without clinically significant side effects.

**Case Presentation:**

We describe a series of three Caucasian women cases that illustrate the efficacy of bilateral ESPB performed at the level of the T7 transverse process to provide intraoperative and postoperative analgesia for laparoscopic gynecological surgery.

**Conclusion:**

Further investigation is recommended to establish the potential for ESPB with dexmedetomidine as adjuvant as an opioid-free anesthetic modality in laparoscopic gynecological surgery.

## Background

Erector spinae plane block (ESPB) is an interfascial block recently performed for thoracic analgesia [1]. The technique can be performed by injecting local anesthetic (LA) in the deep interfascial plane of the erector spinae muscle. This allows wide spread of LA between the erector spinae muscle and the costotransverse process in the paravertebral space, in the intervertebral foramina, in the epidural space and near the ipsilateral sympathetic chain [1]. In 2017, ESPB was described in several case reports for different clinical scenarios, including the management of postoperative pain following open and laparoscopic abdominal surgery and for thoracic and breast surgery [2]. Laparoscopic pain is related to the stretching of the peritoneum and peritoneal irritation caused by the activation of carbon anhydrase in response to insufflation of the parietal peritoneum with carbon dioxide [3]. The use of multimodal anesthesia reduces both intraoperative and postoperative opioids and improves analgesia [2]. The addition of dexmedetomidine to the LA significantly prolongs analgesia, without clinically significant side effects [4]. The use of an epidural catheter is considered a quality standard in abdominal surgery [5], but its placement is not always possible due to technical difficulties or patient-related conditions that contraindicate its insertion. ESPB therefore has the potential to provide both somatic and visceral sensory blocks, which would make it an ideal regional anesthetic technique for laparoscopic abdominal surgery [6]. It is possible to obtain multi-metameric analgesia compared to paravertebral block with a single puncture [7]. Opioid-free analgesia is beneficial in patients with respiratory insufficiency, obstructive sleep apnea syndrome (OSAS), chronic obstructive pulmonary disease (COPD) and in chronic treatment with opioids [8].

## Case presentation

Caucasian women patients consented to the use of their personal data in the publication of this case series for scientific and clinical purposes. They were on chronic opioid treatment for severe pelvic pain. Patients had been taking oxycodone orally for at least 3 months (dose from 15 to 60 mg as prescribed).

Surgery was performed under opioid-free general anesthesia. Premedication included midazolam 0.03 mg/kg and dexamethasone 4 mg intravenously; bilateral ESPB at the T7 level was performed to provide intraoperative analgesia. Propofol (2 mg/kg intravenously) was given for the induction, rocuronium (0.6 mg/kg intravenously) for muscle relaxation, remifentanil (0.1 mcg/kg/minutes) for tracheal intubation, and sevoflurane with minimum alveolar concentration (MAC) 1 for maintenance. The administration of remifentanil was stopped 5 minutes after tracheal intubation. Myo-resolution was monitored by train of four (TOF), and further boluses of 0.2 mg/kg of rocuronium were administered to maintain a TOF = 0. In the awakening phase, sugammadex 4 mg/kg was administered intravenously to revert the neuromuscular block and prevent postoperative residual curarization. For the ESPB, we proceeded with the technique as described by Forero *et al*. [1]. With the patient in sitting position, a high-frequency linear probe (Sonosite HLF 38 × 13, 6 MHz, Fujifilm Sonosite Europe, Amsterdam, Netherlands) was placed in longitudinal orientation at the level of the T7 transverse process (identified under ultrasound [US] guidance starting from the sacrum and going in the cranial direction), 3 cm from the midline. A 21G × 85 mm block needle (Vygon Locoplex) was inserted in plane, with a cranio-caudal direction, until the tip was placed into the plane deep in the erector spinae muscle. After hydro-localization with 3 mL of saline solution to open the plane, 20 mL of 0.5% ropivacaine and dexmedetomidine 0.75 mcg/kg were injected. The procedure was practiced for both sides. Paracetamol 1 g was administered to all patients at 6, 12, 18 and 24 hours after surgery. In the case of acute pain, patients received an analgesic rescue dose according to the visual analog scale (VAS): if VAS ≤ 5, ketorolac 30 mg in 100 mL of saline solution was administered intravenously; if VAS > 5, tramadol 100 mg was administered intravenously. Episodes of postoperative nausea and vomiting (PONV) and postoperative shivering, pruritus, sedation, hemodynamic parameters (heart rate, noninvasive blood pressure, oxygen saturation) and recanalization times were recorded.

### First case

A 28-year-old Caucasian women patient, weight 70 kg, height 170 cm, underwent operative laparoscopy because of left salpingectomy, bilateral ovarian cyst removal, cystic follicle and right corpus luteum excision. She also had several adhesions due to previous surgery that caused pelvic pain. In anamnesis she had endometriosis and nickel allergy. During the operation, no other opioid dose was used. The surgery time was 105 minutes. No surgical complications occurred. The patient did well from an analgesic standpoint, with a VAS score of 0 for the first 24 hours after surgery.

### Second case

A 27-year-old Caucasian women patient, weight 60 kg, height 165 cm, underwent operative laparoscopy because of multiple voluminous uterine myomas. In anamnesis she had hyperprolactinemia and heterozygous mutation of PAI-1. The patient was under cabergoline therapy. During the operation, no other opioid dose was used. The surgery time was 120 minutes. No surgical complications occurred. Fifteen hours after surgery, the patient reported moderate pain (VAS = 3) that was treated with paracetamol 1000 milligrams endovenous; no other analgesics were administered in 24 hours.

### Third case

A 40-year-old Caucasian women patient, weight 60 kg, height 165 cm, underwent operative laparoscopy because of endometriosis. The pain from endometriosis was such that a complete gynecological examination could not be carried out, which required narcosis. In anamnesis she had hypertension pharmacologically controlled and dyslipidemia. During the operation, no other opioid dose was used. The surgery time was 120 minutes; the Trendelenburg position time was 90 minutes. The intra-abdominal pressure was kept higher (15 mmHg) throughout the operation because of no optimal vision of surgical field due to a probable involvement of the left ureter. Nineteen hours after the surgery, the patient reported moderate pelvic pain (VAS = 4) treated with ketorolac 30 mg intravenously in 100 mL of saline solution; no other analgesics were administered in 24 hours.

## Discussion

In a multimodal analgesia strategy, interfascial plane blocks can be a valid alternative and support for opioid-free analgesia. As Beloeil showed in his recent manuscript, patients suffering from chronic pain and/or consuming opioids before surgery benefit from opioid-free anesthesia. These patients are at higher risk of more intense severe postoperative acute pain when consuming more postoperative opioids. It has been shown that all this increases the risk of postsurgical pain chronicization [8].

No patient needed opioids to control intra- or postoperative pain. All patients who are managed daily in the operating room would benefit from this therapeutic strategy. According to Chin *et al*., since the erector muscle of the spine expands from the thoracic to the lumbar region, ESPB provides abdominal analgesia [3]. Studies on cadavers, and by magnetic resonance images, showed that a volume of 20 mL of fluid performed at the T7 transverse process spreads to C7-T2 cranially and L2-L3 caudally [9, 10]. In our opinion, the direction of the needle is fundamental to address the spread of the anesthetic mixture. The best injection point for the mixture is the costotransverse process.

The limit, however, is currently the unpredictability of the diffusion of the mixture despite a correct execution of the technique. Our recent clinical case showed the efficacy and safety of a multidermatomal spread with the use of LA at anesthetic concentrations [11, 12]. Since one of the most common causes of block failure is the injection of the LA into an improper site, that is the rib, in our clinical case the choice of a cranial-caudal approach was well considered before performing the procedure. The position of the patient is quite important for the execution of the block. According to our experience, the sitting position is the best in terms of simplification of the technique. To locate T7, we counted the laminae starting from the sacrum using the US method illustrated by Selvi’s study [13]. Patients reported only low-intensity diffuse abdominal discomfort, probably due to peritoneal irritation related to gas insufflation during the laparoscopy [14], with multi-metameric involvement. In our experience, the intensity of postoperative pain was directly related to the surgical time and the intra-abdominal pressure level of pneumoperitoneum; instead, pain localization is directly related to the kind of pathology and spread of the anesthetic mixture at the caudal lumbar root level, and unpredictable variable. Although epidural analgesia represents the gold standard in abdominal surgery with severe pain, the ESPB safety profile is different from that of epidural analgesia. The ESPB is performed under US guidance, and the target is the transverse process, which is easily identifiable and is relatively distant from neural or major vascular structures [14]. ESPB can also be a valid alternative if there are contraindications to paravertebral block, such as coagulopathy or anticoagulant therapy. ESPB is safer because the target is the transverse process, avoiding accidental pleural punctures; moreover, an advantage is that it provides extensive analgesia with a single puncture [7]. Dexmedetomidine is a potent α2 agonist and is now emerging as an adjuvant to regional anesthesia and analgesia. It can prolong the duration of the nerve block anesthesia when used with LA, and has only a few side effects, resulting in increased effectiveness of the block in terms of duration, less use of opioids and shorter hospital stays, in the absence of clinically significant side effects (bradycardia, hypotension, nausea, vomiting, pruritus) [4, 15, 16]. In some literature studies, the efficacy of dexmedetomidine is comparable to that of dexamethasone, already widely used as an adjuvant in locoregional anesthesia [4].

Some studies indicate that dexmedetomidine, a selective α2-adrenoceptor agonist, may be useful to prevent withdrawal syndrome [17, 18]. In our experience, although limited, there were no episodes of nausea, vomiting, postoperative shiver, pruritus, sedation, bradycardia, hypotension, desaturation or constipation.

## Conclusion

Given our observations, we believe that this approach could be an anesthetic alternative that could be practiced in all patients who take advantage of opioid-free anesthesia either for respiratory problems or for other problems such as chronic use of opioid drugs. Furthermore, this technique can be generalized for all thoracic abdominal surgery. Shorter hospital stays and rapid recanalization could make these interventions suitable for day surgery.

## Data Availability

All data generated or analyzed during this study are included in this published article.

## Abbreviations

COPD: Chronic obstructive pulmonary disease
ESPB: Erector spinae plane block
LA: Local anesthetic
MAC: Minimum alveolar concentration
OSAS: Obstructive sleep apnea syndrome
PAI-1: Plasminogen activator inhibitor-1
PONV: Postoperative nausea and vomiting
TOF: Train of four
US: Ultrasound
VAS: Visual analog scale
